# Comparison of digital PCR platforms using the molecular marker

**DOI:** 10.5808/gi.23008

**Published:** 2023-06-30

**Authors:** Cherl-Joon Lee, Wonseok Shin, Minsik Song, Seung-Shick Shin, Yujun Park, Kornsorn Srikulnath, Dong Hee Kim, Kyudong Han

**Affiliations:** 1 Department of Bio-Convergence Engineering, Dankook University, Yongin 16890, Korea; 2 NGS Clinical Laboratory, Dankook University Hospital, Cheonan 31116, Korea; 3 OPTOLANE Inc., Seongnam 13494, Korea; 4 Department of Microbiology, College of Science & Technology, Dankook University, Cheonan 31116, Korea; 5 Animal Genomics and Bioresource Research Unit (AGB Research Unit), Facult y of Science, Kasetsart University, Bangkok 10900, Thailand; 6 Department of Anesthesiology and Pain Management, Dankook University College of Medicine, Cheonan 31116, Korea; 7 HuNBiome Co., Ltd., R&D Center, Seoul 08507, Korea; 8 Bio-Medical Engineering Research Center, Dankook University, Cheonan 31116, Korea; 9 DKU-Theragen Institute for NGS Analysis (DTiNa), Cheonan 31116, Korea

**Keywords:** digital PCR platforms, molecular marker, point-of-care testing

## Abstract

Assays of clinical diagnosis and species identification using molecular markers are performed according to a quantitative method in consideration of sensitivity, cost, speed, convenience, and specificity. However, typical polymerase chain reaction (PCR) assay is difficult to quantify and have various limitations. In addition, to perform quantitative analysis with the quantitative real-time PCR (qRT-PCR) equipment, a standard curve or normalization using reference genes is essential. Within the last a decade, previous studies have reported that the digital PCR (dPCR) assay, a third-generation PCR, can be applied in various fields by overcoming the shortcomings of typical PCR and qRT-PCR assays. We selected Stilla Naica System (Stilla Technologies), Droplet Digital PCR Technology (Bio-Rad), and Lab on an Array Digital Real-Time PCR analyzer system (OPTOLANE) for comparative analysis among the various droplet digital PCR platforms currently in use commercially. Our previous study discovered a molecular marker that can distinguish Hanwoo species (Korean native cattle) using Hanwoo-specific genomic structural variation. Here, we report the pros and cons of the operation of each dPCR platform from various perspectives using this species identification marker. In conclusion, we hope that this study will help researchers to select suitable dPCR platforms according to their purpose and resources.

## Introduction

Polymerase chain reaction (PCR) is a technique that can amplify target DNA and has applied to research in various fields of biology [1-3]. This technique plays an important role in clinical diagnosis and species identification using molecular markers. It can be applied to various molecular quantification methods in consideration of sensitivity, cost, speed, convenience, and specificity. However, conventional PCR assay have a disadvantage that only qualitative analysis is possible, and quantification is difficult. Therefore, a quantitative real-time PCR (qRT-PCR) that can quantify DNA amplification in real time through fluorescence measurement has been devised [4]. qRT-PCR is used as the gold standard in molecular diagnostics for its high sensitivity, specificity, and accuracy, as well as in forensic biology and medicine [5,6]. However, a qRT-PCR has the following limitations. First, a standard curve should be prepared for absolute quantification as a reference gene expressed at a stable level in various samples. Second, even if a standard curve has been drawn, it must be recreated if a new device or platform used. Finally, qRT-PCR depends on quantification cycle (Cq) values, and quantification is directly affected by PCR inhibitors that distort Cq values [7,8]. Therefore, digital PCR (dPCR) was developed to compensate for these limitations. dPCR is a third-generation PCR capable of real-time absolute quantification without a standard curve [9]. dPCR divides the PCR mixture into independent reactions and expresses digital signals as either a digital signal as a positive droplet “1” or a negative droplet “0” depending on whether amplification occurred or not [10]. The generated droplets can measure the target DNA concentration by the number of copies without a standard curve through Poisson distribution. dPCR has the following advantages compared to qRT-PCR. First of all, quantification is possible without a standard curve. Next, dPCR is possible to detect low copy number targets than the detection limit of qRT-PCR. Finally, dPCR is relatively less affected by PCR inhibitors than qRT-PCR [11,12].

Currently, a variety of commercially available dPCR instruments such as Thermo Fisher Quantstudio 3D, Fluidigm BioMark qdPCR 37K, Formulatrix Constellation, JN Medsys Clarity, QX200 Droplet Digital PCR System (Bio-Rad, Hercules, CA, USA), Raindance Raindrop plus, Stilla Naica, and Lab on an Array (LOAA) are on the market. Among them, we selected the Stilla Naica System (Stilla Technologies, Villejuif, France), Droplet Digital PCR Technology (Bio-Rad), and the LOAA Digital Real-Time PCR Analyzer System (OPTOLANE, Seongnam, Korea) as targets for comparative analysis.

In a previous study, we found the molecular marker that could identify cattle breeds [13]. We investigated the pros and cons of the three dPCR platforms using the Hanwoo-specific molecular marker. This study will help researchers select an appropriate platform according to their purpose and resources in studies applying dPCR.

## Methods

### DNA isolation and PCR

Two hundred microliters of DNA was extracted from 9 Hanwoo and 9 Holstein blood samples (200 μL) using Exgene Blood SV mini kit (GeneAll Biotechnology, Seoul, Korea). All research protocols and animal experiments in this study were reviewed and approved by the Dankook University Institutional Animal Care and Use Committee (DKU IACUC) in South Korea (DKU-22-055). The PCR composition performed to verify the molecular marker (DEL_96) in all samples is as follows; PCR amplification was carried out using 10 ng and 20 ng of template, 1 μL of DNA, 7 μL of D.W, 1 μL of each oligonucleotide primer of 200 nM, and 10 μL of BioFACT Lamp Taq DNA Polymerase (BioFACT, Daejeon, Korea) in a total volume of 20 μL (Table 1). PCR amplification was performed by following process: pre-denaturation step of 5 min at 95°C, followed by 35 cycles of denaturation step 30 s at 95°C, annealing step of 40 s at 60°C, and extension step of 1 min at 72°C, followed by a final extension step of 10 min at 72°C.

### Stilla Naica System for Crystal Digital PCR

The droplet digital PCR (ddPCR) reaction mixture consisted of 5 μL of Naica multiplex PCR Mix Buffer A (5×, cat. No. R10052, Stilla Technologies), 1 μL of Naica multiplex PCR Mix Buffer B 4% (100%, cat. No. R10052, Stilla Technologies), 1 μL of FAM probe/primer mix 25×, 1 µL of VIC probe/primer mix 25×, 50 ng DNA, and nuclease-free water up to 25 µL. The reaction mixture was loaded onto a Sapphire Chip (Stilla Technologies), and sample partitioning and thermal cycling were performed on the Naica Geode. The ddPCR was carried out in the following steps: initial denaturation step of 3 min at 9°C, 15s at 60°C for annealing and extension, with a release step that lowers the pressure and temperature for 33 min. Each sample produced 20,000 to 30,000 droplets. The fluorescence of the Sapphire Chip, where PCR was completed, was measured using Naica Prism 3 equipment, and the fluorescence value extracted for each droplet was analyzed using Crystal Miner software (Stilla Technologies) [14].

### Bio-Rad for QX200 Droplet Digital PCR System

The ddPCR reaction mixture contained 10 μL of 2× ddPCR supermix for probes (No dUTP) (#186-3024, Bio-Rad), the final concentration of 250 nmol/L for each of the probes, 450 nmol/L for the forward and reverse primers, 50 ng DNA, and nuclease-free water up to 20 µL. ddPCR reaction mixture was dispensed into the middle line in the DG8 cartridge (#186-4008, Bio-Rad), and 70 μL of generation oil was dispensed into the bottom wells. The sample is split into approximately 20,000 water-oil emulsion droplets using the QX200 Droplet generator. Forty microliters of the resulting water-oil emulsion droplets were transferred to a 96-well plate sealed with PX1 PCR plate Sealer (Bio-Rad). The QX200 was carried out in the following steps: enzyme reaction step of 3 min at 50℃, initial denaturation step of 15 min at 95℃, followed by 40 cycles of denaturation 10 s at 95℃, 20 sec at 60℃ for annealing and extension. Droplets were analyzed using QuantaSoft version 1.7.4.0917 (Bio-Rad) [15,16].

### OPTOLANE for LOAA Digital PCR System

The reaction mixture consisted of 10 μL 3× Dr. PCR Master Mix (OPTOLANE), 10 µL Primer & Probe mix (final concentration of 20 pmol for the forward and reverse primer, 10 pmol for each of the probes), and 10 µL of DNA containing 50 ng. The reaction mixture was placed in a semiconductor-based cartridge and spread evenly on the chip using a sample loader, LOAA POSTMAN. The LOAA dPCR system was carried out in the following steps: enzyme reaction step of 3 min at 50 ℃, initial denaturation step of 15 min at 95℃, followed by 40 cycles of denaturation 10 s at 95℃, 20 s at 62 ℃ for annealing and extension. The PCR-completed chip was automatically analyzed with LOAA Dr. PCR software 3.0.0 (OPTOLANE) [17,18].

## Results and Discussion

**Fig. 1.**
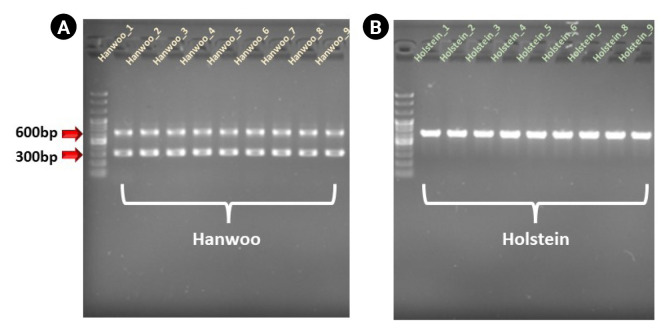
Polymorphic patterns of the Del_96 region in 9 Hanwoo (A) and Holstein (B) genomes. As a result of gel chromatography, heterozygous alleles at 680 bp and 310 bp were identified in the 9 Hanwoo samples, but no deleted alleles were confirmed in the Holstein sample.

A previous study reported that the Hanwoo-specific molecular marker was found only in the Hanwoo genome by nonallelic homologous end-joining [19]. Therefore, it can be used as a strong molecular marker to differentiate between Hanwoo and Holstein. As shown in Fig. 1, the Hanwoo strain has a polymorphic structure in the Del_96 region by a TE-association deletion event. Among various commercial dPCR equipment, we selected the Stilla Naica System (Stilla Technologies), Droplet Digital PCR Technology (Bio-Rad), and the LOAA Digital Real-Time PCR Analyzer System (OPTOLANE) as targets for comparative analysis. As shown in Table 2, Crystal Digital PCR takes about 2.5 h, which is relatively faster than QX200, because droplet formation and PCR proceed in Geode equipment. In the QX200, it took about 6 h, the longest time, from droplet formation in the Droplet Generator to amplification in the PX1 PCR Cycler to finally obtain the result. However, it has the advantage of being able to perform dPCR for more samples at once than Crystal Digital PCR and LOAA dPCR [20]. Unlike two equipments, LOAA dPCR can obtain experimental results in the shortest time as both PCR and data analysis are performed in a single equipment in a chip format. In addition, OPTOLANE's unique technology can be applied to the chip to reduce the weight and size of the equipment, so it is highly applied to point-of-care testing. However, in the case of LOAA dPCR equipment, there is a disadvantage in that dPCR can be performed on only one sample per cartridge.

**Fig. 2.**
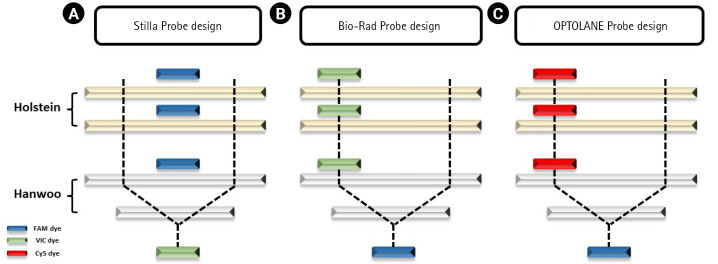
Probe designs for each equipment. (A) The FAM dye was designed to detect all bovine genomes through digital PCR analysis, and the VIC dye was designed at the Del_96 boundary to detect only Hanwoo. (B) The VIC dye was designed to detect all cattle genomes, and the FAM dye was designed to detect only Hanwoo at the Del_96 boundary. (C) The Cy5 dye was designed to detect all cattle genomes, and the FAM dye was designed to detect only Hanwoo at the Del_96 boundary of Hanwoo.

**Fig. 3.**
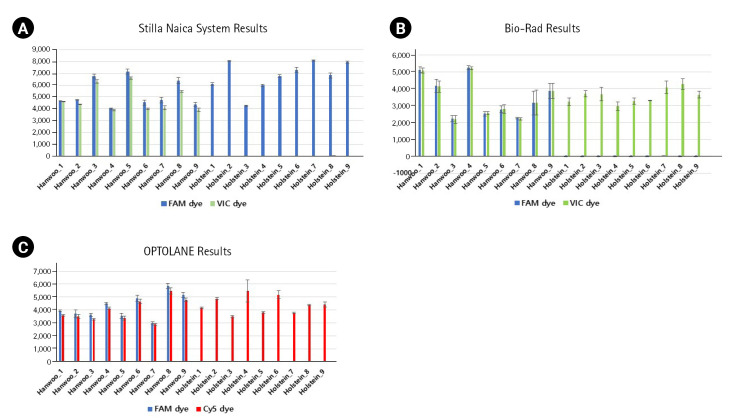
Comparison of each positive droplet rate through three repeated experiments. (A) X-axis and Y-axis show the name of each sample and the number of positive droplets formed with FAM dye and VIC dye using the Stilla Naica System. (B) X-axis and Y-axis indicate the name of each sample and the number of positive droplets formed with FAM dye and VIC dye using Bio-Rad equipment. (C) X-axis and Y-axis indicate the name of each sample and the number of positive droplets formed with FAM dye and Cy5 dye using OPTOLANE equipment.

**Fig. 4.**
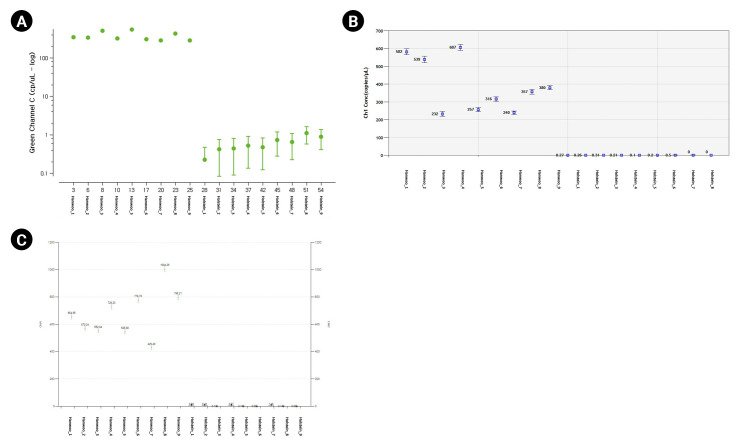
Absolute copy number comparison of each equipment using Hanwoo-specific probe. The X-axis represents sample information, Y-axis represents concentration (copy/μL). (A–C) The fluorescence of the Hanwoo-specific probe was detected in Hanwoo but not in Holstein.

For the dPCR analysis of the three instruments, 9 Hanwoo and 9 Holstein blood samples were used as DNA templates, and three repetitions were performed with the FAM probe (Thermo Fisher Scientific, Waltham, MA, USA), VIC probe (Thermo Fisher Scientific), Cy5 probe (SFC Probes, Cheongju, Korea) customized for each manufacturer (Fig. 2, Supplementary Tables 1, 2). The Crystal Digital PCR formed 20,000 and 30,000 droplets, producing about 23,243 on average. FAM dye was detected in all Hanwoo and Holstein genomes, and VIC dye was only detected in Hanwoo samples (Fig. 3). The signal of the VIC dye was detected as an average 367.25 channel concentration (copy/µL) in the genome of Hanwoo. The QX200 can form 20,000 droplets and produce about 13,409 droplets on average. FAM dye was used as a Hanwoo-specific probe, and VIC dye was designed to fit the QX200 instrument to be detected in all genomes of Hanwoo and Holstein. VIC dye was detected in all genomes of Hanwoo and Holstein, and FAM dye was detected only in Hanwoo samples. Signals from the FAM dye was detected in average 376.96 channel concentration (copy/µL) in the genome of Hanwoo. LOAA dPCR can form 20,000 droplets, the same as QX200, and generate about 16,715 on average. FAM dye was used as a Hanwoo-specific probe, and Cy5 dye was designed to detect all genomes of Hanwoo and Holstein. Cy5 dye was detected in all samples like other instruments, and FAM dye was detected only in Hanwoo samples. The FAM dye signal was detected as an average 681.73 concentration (copy/µL) in the genome of Hanwoo. As a result, Hanwoo-specific probes in all dPCR instruments showed significant detection only in Hanwoo samples, suggesting that all Hanwoo samples contained a specific deletion region (Fig. 4). However, in Holstein samples, Crystal Digital PCR, QX200, and LOAA dPCR using a Hanwoo-specific probe showed channel concentrations (copy/µL) of 0.66, 0.19, and 0.35, respectively. This result is consistent with previous studies: (1) Probes designed in the TE region can detect non-specific signals in structure variation with high similarity. (2) Abnormally high fluorescence intensity measured in dPCR analysis can be mistakenly recognized as a positive well. Nevertheless, the Hanwoo-specific probe was statistically sufficient to discriminate between Hanwoo and Holstein.

Since the outbreak of the coronavirus disease-19 pandemic, the need for equipment that complements conventional PCR, which can only perform qualitative analysis, and qRT-PCR, which is greatly affected by PCR inhibitors, has emerged. Species identification experiment results using the Stilla Naica System, Droplet Digital PCR Technology, and LOAA Digital Real-Time PCR Analyzer System among commercially available dPCRs confirmed that all equipment has the potential as a platform for species identification. In particular, compared to other dPCR equipment, the LOAA Digital Real-Time PCR Analyzer System has been made smaller and lighter with the manufacturer's proprietary technology. dPCR has operational pros and cons depending on each platform. In addition, consumables of dPCR in common are expensive compared to qRT-PCR. However, dPCR can contribute to developing genetically modified organism testing and drug resistance research in addition to species identification with high accuracy and sensitivity. Therefore, this study is expected to help researchers select a suitable dPCR platform according to their purpose and resources.

## Figures and Tables

**Table 1.** Cattle gDNA quality control and dsDNA concentration

Sample No.	Slaughter No.	Breed	Target size (bp)	Date of slaughter	Spectrometer		
					DNA concentration (ng/μL)	A260/A280	A260/A230
Hanwoo_1	10	Hanwoo	680/310	13 Jul 2015	31.7	1.84	1.8
Hanwoo_2	11	Hanwoo	680/310	6 Jul 2015	39.83	1.84	1.59
Hanwoo_3	13	Hanwoo	680/310	13 Jul 2015	35.47	1.84	1.65
Hanwoo_4	16	Hanwoo	680/310	6 Jul 2015	23.66	1.92	1.61
Hanwoo_5	20	Hanwoo	680/310	13 Jul 2015	39.31	1.87	1.43
Hanwoo_6	21	Hanwoo	680/310	6 Jul 2015	22.92	1.87	1.75
Hanwoo_7	243	Hanwoo	680/310	6 Jul 2015	35.52	1.88	2.09
Hanwoo_8	256	Hanwoo	680/310	6 Jul 2015	27.32	1.82	1.26
Hanwoo_9	282	Hanwoo	680/310	6 Jul 2015	41.78	1.88	2.22
Holstein_1	264	Holstein	680 (only)	6 Jul 2015	33.25	1.81	1.83
Holstein_2	274	Holstein	680 (only)	6 Jul 2015	23.55	1.84	1.76
Holstein_3	280	Holstein	680 (only)	6 Jul 2015	19.53	1.86	1.71
Holstein_4	292	Holstein	680 (only)	6 Jul 2015	67.14	1.86	2.21
Holstein_5	296	Holstein	680 (only)	6 Jul 2015	26.86	1.73	1.11
Holstein_6	306	Holstein	680 (only)	6 Jul 2015	35.22	1.82	2.21
Holstein_7	317	Holstein	680 (only)	6 Jul 2015	19.2	1.67	1.14
Holstein_8	320	Holstein	680 (only)	6 Jul 2015	27.57	1.75	1.11
Holstein_9	332	Holstein	680 (only)	6 Jul 2015	33.57	1.77	1.04

**Table 2.** Comparison of three different dPCR platforms

	Stilla	Bio-Rad	OPTOLANE
	Crystal Digital PCR	QX200 Droplet Digital PCR	Lab on an Array digital PCR
dPCR type	Droplet	Droplet	Chip
Detection mode	End point	End point	Real-time
Partitions	30,000	20,000	20,000
Duration	~2.5 h	~6 h	~1.5
Detection	3 colors	2 colors	2 colors
Price	×1.1	×1	×0.2

dPCR, digital polymerase chain reaction.

## Supplementary Materials

Supplementary Table 1. Triplication results using digital PCR.

Supplementary Table 2. Information of digital PCR (dPCR) primer and probe sequences.
