# Supplementary material for: Comparison of digital PCR platforms using the molecular marker
Source: Genomics Inform. 2023 Jun 30;21(2):e24. doi: 10.5808/gi.23008 (PMC10326530; doi:10.5808/gi.23008)
Supplement: Supplementary Table 1. — Triplication results using digital PCR. [file gi-23008-Supplementary-Table-1.pdf]

Supplementary Table 1. Triplication results using digital polymerase chain reaction

Chamber name	No. of droplets	Blue channel			Green channel		
		C (cp/μL)	No. of Pos	Blue average	C (cp/μL)	No. of Pos	Green average
Hanwoo_1	25,040	331.7	4,679	4,667	330	4,657	4,615
	24,759	333.1	4,644		327.7	4,576	
	23,901	349.4	4,679		343.7	4,611	
Hanwoo_2	23,841	353.2	4,713	4,765	323.8	4,359	4,374
	23,983	359	4,811		320.3	4,342	
	23,350	366.4	4,770		336.7	4,422	
Hanwoo_3	24,314	557.3	7,138	6,763	509.5	6,618	6,310
	22,838	541.9	6,549		507.4	6,195	
	22,191	566.2	6,602		517	6,116	
Hanwoo_4	21,698	323.7	3,966	4,015	316.5	3,886	3,884
	22,708	323	4,143		311.7	4,012	
	21,929	317.2	3,936		301	3,753	
Hanwoo_5	22,751	561.7	6,723	7,142	541.4	6,519	6,609
	23,240	599.4	7,248		525.9	6,498	
	23,426	614.2	7,454		550.8	6,810	
Hanwoo_6	24,616	318.7	4,437	4,527	284.7	4,005	3,984
	22,383	332.2	4,188		303.6	3,861	
	24,114	368.9	4,956		297.7	4,086	
Hanwoo_7	24,820	375.5	5,182	4,715	313.3	4,405	4,081
	23,826	341.5	4,570		282.5	3,849	
	22,840	342.5	4,393		307.8	3,989	
Hanwoo_8	24,704	526	6,908	6,381	400.5	5,460	5,496
	24,338	480.7	6,304		422.4	5,636	
	23,866	458.1	5,931		410.7	5,393	
Hanwoo_9	25,959	319	4,683	4,360	281.6	4,181	3,928
	25,340	291.9	4,217		269.8	3,924	
	23,129	319.6	4,180		277.8	3,679	

Holstein_1	21,701	517.8	5,989	6,095	0.22	3	6
	21,501	519.4	5,949		0.6	8	
	23,576	502.9	6,347		0.54	8	
Holstein_2	23,128	691.2	8,099	8,017	0.42	6	5
	23,025	686.7	8,021		0.28	4	
	23,658	654.7	7,930		0.34	5	
Holstein_3	21,659	357.7	4,330	4,266	0.44	6	5
	21,678	343.6	4,181		0.44	6	
	22,480	339.3	4,287		0.21	3	
Holstein_4	21,759	500.2	5,831	5,984	0.52	7	3
	22,889	479.8	5,919		0.07	1	
	23,025	503.2	6,202		0.14	2	
Holstein_5	22,811	548.8	6,611	6,743	0.56	8	7
	22,996	546.4	6,640		0.35	5	
	23,959	552.1	6,979		0.47	7	
Holstein_6	22,223	620.7	7,133	7,274	0.65	9	10
	23,229	645.5	7,698		0.83	12	
	22,109	609.5	6,991		0.73	10	
Holstein_7	22,157	736.2	8,157	8,069	0.72	10	13
	22,951	690.7	8,032		0.84	12	
	22,226	717.6	8,019		1.3	18	
Holstein_8	23,986	554.2	7,009	6,815	0.74	11	23
	23,260	517.1	6,411		2.83	41	
	24,715	536.3	7,026		1.1	17	
Holstein_9	22,015	701.2	7,798	7,937	0.8	11	12
	23,015	680.9	7,963		0.77	11	
	23,473	673.5	8,050		0.89	13	
