# Supplementary material for: Comparison of digital PCR platforms using the molecular marker
Source: Genomics Inform. 2023 Jun 30;21(2):e24. doi: 10.5808/gi.23008 (PMC10326530; doi:10.5808/gi.23008)
Supplement: Supplementary Table 2. — Information of digital PCR (dPCR) primer and probe sequences. [file gi-23008-Supplementary-Table-2.pdf]

Supplementary Table 2. Information of digital polymerase chain reaction primer and probe sequences

Paltform	Name	Sequence
Stilla	BTWT-F[a]	AGTTCATGTCCACTGCATTGGT
	BTWT-R	GGAGAAGAGAGCATCAGCAGATG
	BTWT-P	**/FAM/**ATGCCATCAGCCATC**/MGB/**
	BTdel-F[b]	TTCATATATTTTGTGATTGACTGTCCTATC
	BTdel-R	TGTGGTGCTGGACAAGATTCC
	BTdel-P	**/VIC/**CTTGCTTTTCCCTCCTT**/MGB/**
Bio-Rad	BTWT-F	CTTGCTTTTCCCTCCAAGACA
	BTWT-R	GTCGACTCGTTGGAAAAGTTCCT
	BTWT-P	**VIC**/CTTCTCCTCTGCCCTCAATCTTTCCTAGCA/**SFCQ1**
	BTdel-F	CCTTGCTTTTCCCTCCTTGG
	BTdel-R	CGTGTACAAGTGTGAGAGCTGAACTGT
	BTdel-P	**/FAM/**CACAGTTTGAAGTCATCAATTCTTTGGCGCT/**SFCQ1/**
OPTOLANE	BTWT-F	CTTGCTTTTCCCTCCAAGACA
	BTWT-R	GTCGACTCGTTGGAAAAGTTCCT
	BTWT-P	/**SFC620**/AGT/FAM/CTTCTCCTCTGCCCTCAATCTTTCCTAGCA/**SFCQ2**
	BTdel-F	CCTTGCTTTTCCCTCCTTGG
	BTdel-R	CGTGTACAAGTGTGAGAGCTGAACTGT
	BTdel-P	**/FAM/**CACAGTTTGAAGTCATCAATTCTTTGGCGCT/**SFCQ1/**

[a]BTWT (Bos Taurus Wild Type) is found in both Hanwoo and Holstein.

[b]BTdel (Bos Taurus deletion) is found only in Hanwoo.’
